# NOX proteins and ROS generation: role in invadopodia formation and cancer cell invasion

**DOI:** 10.1186/s40659-024-00577-z

**Published:** 2024-12-19

**Authors:** Nelson Quilaqueo-Millaqueo, David A. Brown-Brown, Jetzabel A. Vidal-Vidal, Ignacio Niechi

**Affiliations:** Instituto de Bioquímica y Microbiología, Facultad de Ciencias, Universidad Austral de Chile, 5090000 Valdivia, Chile

**Keywords:** NOX, ROS, Invadopodia, Cancer cell invasion

## Abstract

NADPH oxidases (NOX) are membrane-bound proteins involved in the localized generation of reactive oxygen species (ROS) at the cellular surface. In cancer, these highly reactive molecules primarily originate in mitochondria and via NOX, playing a crucial role in regulating fundamental cellular processes such as cell survival, angiogenesis, migration, invasion, and metastasis. The NOX protein family comprises seven members (NOX1-5 and DUOX1-2), each sharing a catalytic domain and an intracellular dehydrogenase site. NOX-derived ROS promote invadopodia formation, aberrant tyrosine kinase activation, and upregulation of matrix metalloproteinases (MMPs). Specifically, NOX5 modulates adhesion, motility, and proteolytic activation, while NOX1 likely contributes to invadopodia formation and adhesive capacity. NOX2 and NOX4 are implicated in regulating the invasive phenotype, expression of MMPs and EMT markers. DUOX1-2 participate in epithelial-mesenchymal transition (EMT), crucial for invasive phenotype development. Soluble molecules such as TGF-β and EGF modulate NOX protein activation, enhancing cell invasion through localized ROS production. This review focuses on elucidating the specific role of NOX proteins in regulating signaling pathways promoting cancer cell spread, particularly EMT, invadopodia formation and invasive capacity.

## Background

Reactive oxygen species (ROS) are molecules derived from the reduction of molecular oxygen (O_2_). After oxidizing carbon in biomolecules, this process generates a range of oxidant species, including superoxide anion, hydroxyl radical, peroxyl radical, and peroxide [1]. In cancer cells, ROS play a crucial role in fundamental processes by interacting with different signaling pathways involved in cellular survival, angiogenesis, tumor progression, and metastasis [2–5]. ROS levels in cancer cells are higher compared to non-tumor cells, primarily due to their elevated metabolic rates. The main sources of ROS in cancer cells are mitochondria and the enzymatic activity of NADPH oxidase proteins (NOXs). The heightened presence of ROS contributes to the dynamic cellular environment in cancer, influencing key processes that drive malignancy [4]. However, the exact contributions of ROS from specific sources, such as mitochondria versus NOXs, in cancer progression remain poorly studied.

The NOX protein family is considered one of the most crucial sources of ROS production in eukaryotic cells. This family comprises seven members known as NOX1-5 and DUOX1-2, each participating in several cellular processes, including hormone production, modification of the extracellular matrix, host defense for immune cells, and redox signaling [6–8]. NOXs, membrane proteins with a conserved catalytic core of six transmembrane alpha helices, display structural similarity but vary in cellular distribution, activation mechanisms, and regulatory systems [9, 10]. Recent studies have underscored the significant importance of NOX-derived ROS in cancer cells, showcasing their crucial influence on cell survival, tumor development, and progression [11, 12]. While there is substantial evidence supporting the role of NOX-derived ROS in cancer, the molecular mechanisms linking NOX activity to tumor progression remain incompletely understood.

This involvement occurs via the activation of different signaling pathways related to the expression and secretion of matrix metalloproteinases (MMPs) [13, 14], the regulation of epithelial-mesenchymal transition, and the generation of invadopodia [15]. This literature review aims to compile relevant background information on the influence of the NOX protein family expression and function on the formation and activity of invadopodia, as well as their role in cell invasion.

### Cell invasion and invadopodia

The fundamental process enabling cancer cells to migrate or invade, involves the acquisition of mesenchymal characteristics, which occurs through epithelial-mesenchymal transition (EMT). EMT is characterized by the loss of epithelial features, such as cellular polarity and cell–cell junctions, leading to the acquisition of a mesenchymal phenotype associated with increased cell motility [16]. This transition involves the loss of molecular epithelial markers and the acquisition of mesenchymal markers such as N-cadherin, Vimentin, and fibronectin and the loss of E-cadherin [17]. Migration in cancer cells is mediated by the coordinated action of cytoskeletal components and proteins associated with the formation of protrusions, such as lamellipodia and filopodia [18]. The EMT process and the acquisition of increased cell motility are crucial for tumor cells to invade healthy tissues.

The cell invasion is the capacity through which tumor cells can penetrate and invade surrounding tissues until they reach the lymphatic and blood vessels, facilitating dissemination to more distant organs [19–21]. This process represents the initial step of metastasis and is mediated by various biochemical and physical factors in the tumor microenvironment, inducing a remodeling of the cytoskeleton and extracellular matrix (ECM) [22]. To initiate the invasion process, tumor cells must penetrate the ECM through the formation of specialized protruding structures known as invadopodia [23]. Invadopodia are composed of actin and surrounded by several adhesion proteins such as integrins, paxillins, and talins [24, 25]. The initiation of invadopodia formation involves the creation of precursor complexes enriched with actin-regulating proteins like cortactin, cofilin, WASP, and Arp2/3. These complexes enable cells to create protrusions by assembling actin filaments, which gradually become organized and anchored to the cell's surface, this, together with the recruitment of several proteins, ultimately forming functional structures [24, 26]. This activity is primarily mediated by metalloproteinases (MMPs), a family of zymogen proteases involved in the degradation and remodeling of the ECM [24–27]. MMPs can be anchored to the plasma membrane or secreted into the extracellular medium [27]. Studies have demonstrated that NOX-dependent ROS play a key role in this process. However, much of what is known about invadopodia formation is based on simplified in vitro models, which may not fully reflect the complexity of ROS dynamics and cellular interactions in vivo*.*

### Structural features of NADPH oxidases and their role in ROS production

**Fig. 1:**
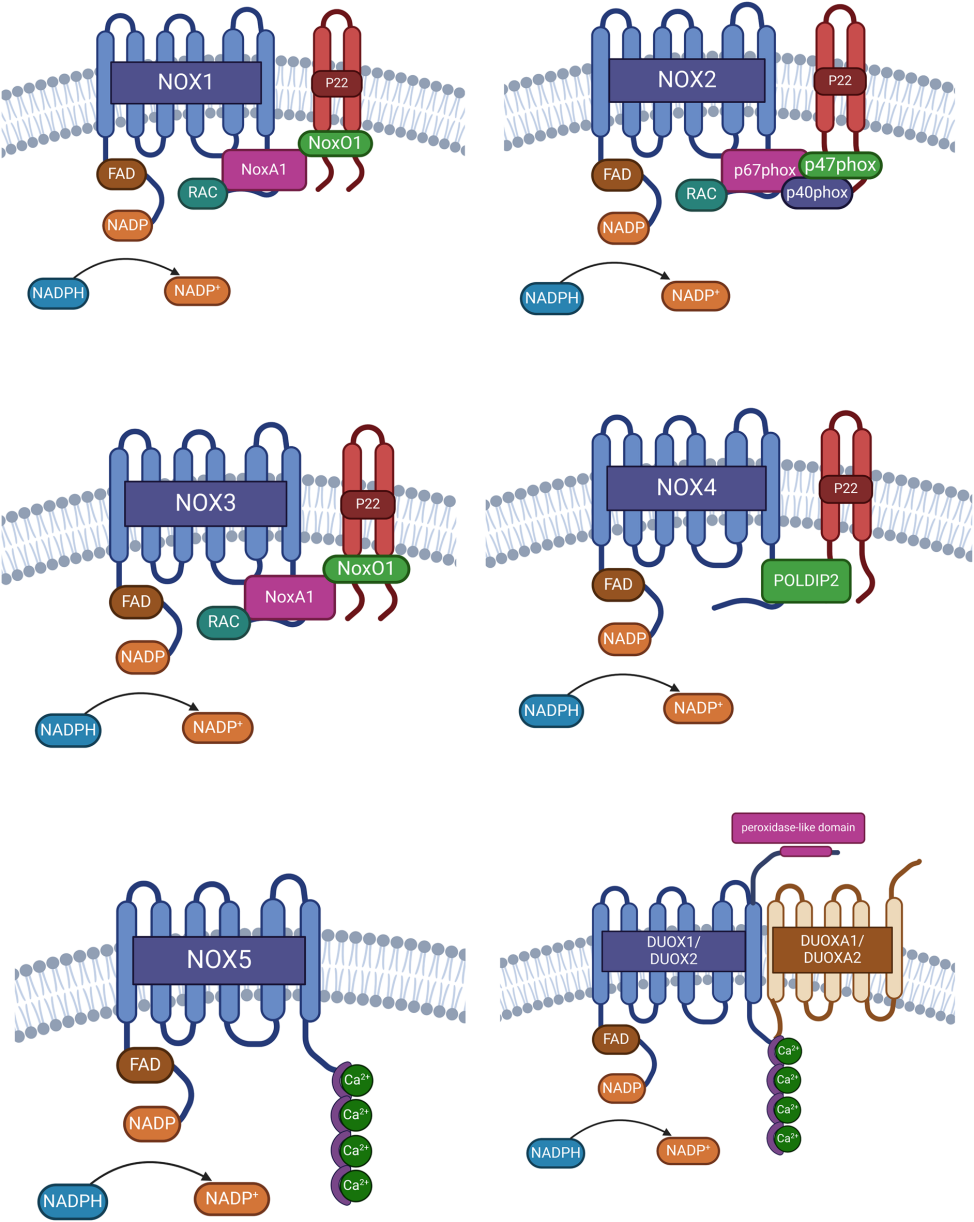
Structural components of active NOX and DUOX enzymes. Key elements that form the active NOX and DUOX enzymes, which are crucial for the generation of reactive oxygen species (ROS). NOX1, NOX2, and NOX3 are shown to share common structural features and rely on cytosolic subunits for their activation. In contrast, NOX4 is depicted as being constitutively active, although it has been described as further modulated by interaction with POLIDIP2. The figure also highlights that NOX5 and the DUOX isoforms respond to changes in intracellular calcium concentrations

NADPH oxidases (NOX) constitute a family of membrane proteins and serve as one of the main sources of reactive oxygen species (ROS) in eukaryotic cells [28]. NOX proteins can be classified based on the type of ROS production. Specifically, NOX1-3 and NOX5 are known as superoxide anion (O₂⁻) producers, while NOX4 and DUOX1-2 are mainly related with the production of H₂O₂ [29]. ROS generation by NOXs usually involves the oxidation of NADPH molecules, wherein the electron from NADPH is transferred to a molecule of oxygen, resulting in the production of O₂⁻ and NADP + [30–34]. O₂⁻ is rapidly converted to hydrogen peroxide (H₂O₂) by the action of the antioxidant enzyme Superoxide Dismutase (SOD) through a two-step dismutation reaction. In the first step, O₂⁻ binds to the active site of SOD, where it transfers an electron to the SOD metal cofactor, leading to its reduction. This electron transfer disrupts the bonds between the metal cofactor and nearby histidine residues, inducing protonation of histidines and facilitating the release of molecular oxygen as the first product. In the second step, a new O₂⁻ binds to SOD's active site, receiving the electron from the previously reduced metal cofactor. This electron transfer promotes the protonation of the new O₂⁻, ultimately generating H₂O₂ as the final product and restoring the bonds between the metal cofactor and histidines in SOD [30, 31]. On the other hand, NOX4 directly produces H₂O₂, utilizing 90% of the electron flow, while the remaining 10% of the electron flow is used for O₂⁻ production [35]. For H₂O₂ formation, two electrons are sequentially transferred to an O₂ molecule, resulting in its double reduction. In the first reduction step of O₂ generates an O₂⁻ molecule, which interacts with the heme group of NOX4, forming a stable intermediate between the heme group and O₂⁻. This interaction facilitates a second reduction of O₂⁻, followed by protonation, leading to the formation of H₂O₂ [35, 36]. It has been proposed that H₂O₂ generation by DUOX1 and DUOX2 proteins occurs through the reduction of two O_2_ molecules, producing two O₂⁻ molecules. These O₂⁻ then undergo a dismutation reaction, leading to the formation of H₂O_2_ [37]. Despite the well-established role of NOX proteins in ROS production, the exact biochemical and molecular processes leading to their activation remain poorly understood, particularly with respect to isoform-specific functions in cancer cell invasion. Structurally, NOX proteins are composed of four transmembrane domains (TM) and an intracellular dehydrogenase domain (DH) [9, 29]. The TM consists of six transmembrane alpha helices connected by intra- and extracellular loops. Additionally, this domain is associated with two heme groups responsible for electron transfer to oxygen [10]. The DH contains binding sites for NADPH substrate, and FAD, facilitating electron transfer to the heme groups at TM domain [10, 29]. However, current research often overlooks how differences in the cellular localization and regulation of NOXs might influence their contributions to ROS production in different cellular contexts. Notably, the activation of NOXs at the membrane requires the formation of distinct complexes specific to each NOX isoform, as illustrated in Fig. 1. NOX1-4 proteins are associated with a membrane-associated protein, p22phox, which acts as a scaffold protein for the maturation and folding of the active NOX protein. Furthermore, for NOX1-3, p22phox functions as a platform for the binding of cytosolic activator proteins [10, 38]. In the case of NOX5, this variant does not form a complex with p22phox, and conversely, for DUOX1 and DUOX2, the presence of scaffold proteins DUOXa1 and DUOXa2, respectively, is imperative for their activation [38]. For NOXs requiring the recruitment of cytosolic proteins, the specific proteins involved vary depending on the type of NOX, in the case of NOX1/p22phox, the adapter protein NoxO1 is recruited, facilitating the binding of the activator protein of NOX1 (NoxA1) and Rac, which then activate the catalytic domain of NOX1. Conversely, NOX2/p22phox utilizes the adapter protein p47phox, enabling the binding of activator proteins p67phox and Rac [10, 38]. For NOX3, its low expression has hindered the determination of interacting proteins for activation, but it is suggested that its activation depends on NoxO1 and NoxA1, similar to NOX1 [24, 38]. Finally, while NOX4/p22phox acts constitutively, its activity is potentiated through the binding of the protein POLDIP2 [38, 39]. The formation of regulatory complexes controlling NOX activity at the membrane level generates localized ROS, for example, at invadopodia, thereby linking to the invasive capacity of tumor cells. Despite extensive studies on NOX activators, the complexities of their interactions with different subunits and activators have not been fully addressed, leaving gaps in our understanding of their precise regulatory mechanisms.

### NOX-derived ROS in invadopodia function and cancer cell invasion

**Fig. 2:**
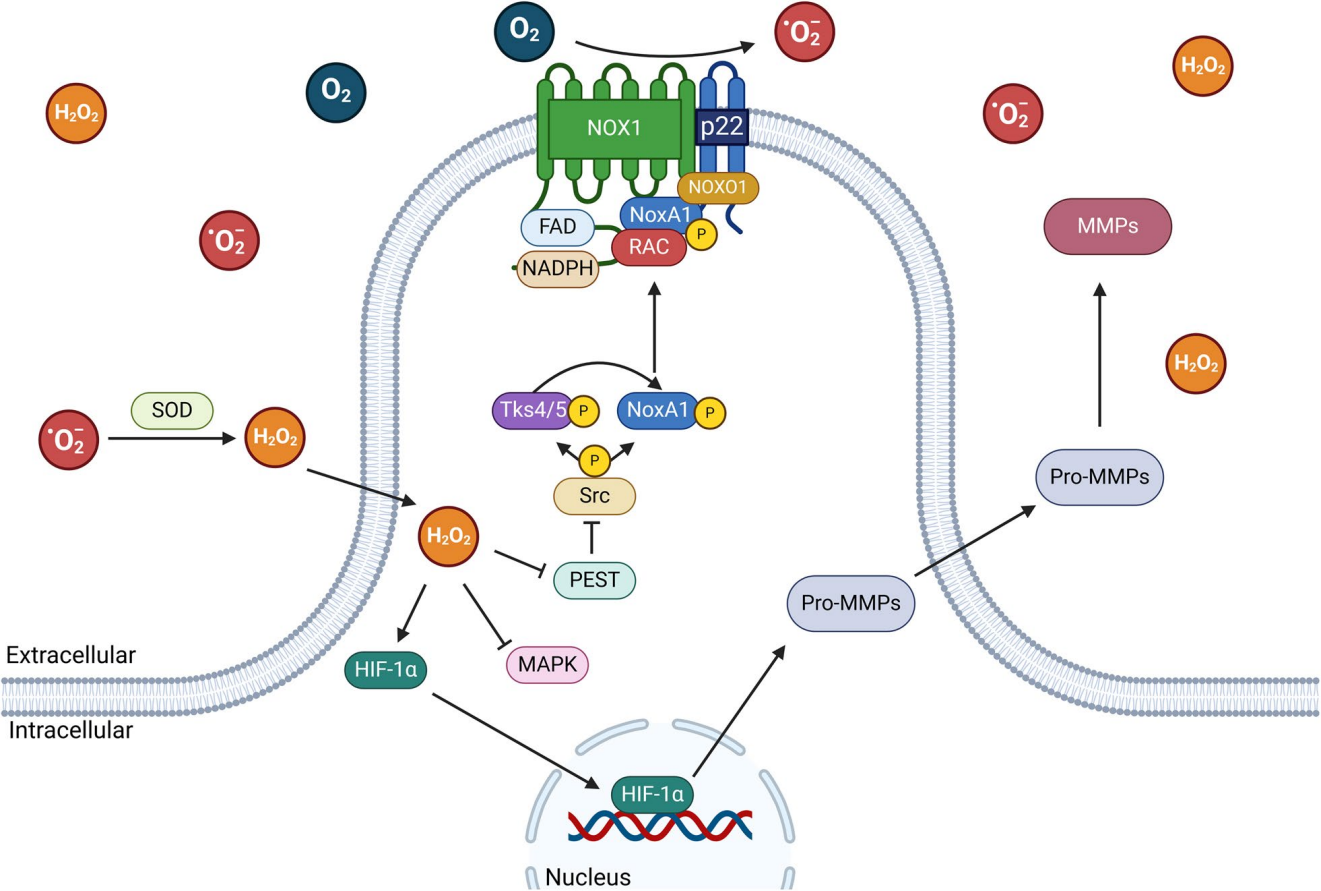
Activation and function of NOX1 in invadopodia formation. Activation mechanism of NOX1 within invadopodia and its contribution to the localized production of reactive oxygen species (ROS). The figure highlights the formation of the NOX1 complex, which includes the recruitment of the cytosolic activator proteins NoxO1, NoxA1, Rac, and the p22phox scaffold protein. The SRC-dependent phosphorylation of Tks4 and Tks5 facilitates the interaction with NoxA1, leading to NOX1 activation and ROS production. These ROS play a crucial role in the formation and function of invadopodia, enabling cancer cell invasion by enhancing the proteolytic activity of matrix metalloproteinases (MMPs) within invadopodia. The figure also shows the overall importance of NOX1 in modulating the invasive phenotype in cancer cells

Localized ROS production by NOX proteins is crucial in invadopodia formation [40, 41]. Studies in melanoma have shown an increase in localized ROS within invadopodia, suggesting that this rise is due to the activity of NOX proteins present in these cellular structures [42]. In colon cancer, the presence of the NOX1 protein in invadopodia has been observed, and its inhibition impair invadopodia formation, suggesting that NOX1 and ROS production are essential for the formation of the invasive phenotype in these cancer cells [43, 44]. The activation of NOX1 in colon cancer is mediated by the SRC-dependent phosphorylation of Tks4 and Tks5, which interacts with NoxA1 (Fig. 2) [43–47]. In the case of NOX2, the activation mechanism is different because Tks proteins do not interact with the activator protein p67phox [46, 47]. NOX4 has been detected in invadopodia alongside F-actin, although the constitutive regulation mechanism of NOX4 remains unclear [42, 48]. In lung cancer, it has been discovered that DUOX1 is epigenetically silenced through DNA hypermethylation in its promoter region, leading to an increased expression of molecular markers associated with EMT [49]. On the other hand, in colon cancer, DUOX2 has been observed to increase ROS production due to elevated DUOX2 protein levels, which, in turn, promotes the expression of EMT-associated markers [50]. In colon cancer, NOX5 participates in the regulation of integrin-linked kinase signaling pathways, which are involved in cell adhesion and movement, correlating with the motility of tumor cells [51]. In breast cancer, it was observed that the expression of NOX5 is regulated by the transcription factor STAT5A, and depletion of NOX5 leads to a reduction in the invasive capacity of tumor cells [52]. In prostate cancer, increased expression of NOX5 is associated with elevated ROS levels and enhanced invasive and proteolytic capacity through the activation of HIF-1α and an increase in MMP14 levels [53, 54]. The increase in ROS induced by NOX proteins has been observed to stabilize HIF1α [55], this is mediated by the oxidation of cysteine residues present in the prolyl hydroxylase domain-containing protein 2 (PHD2), which is responsible for inactivating HIF-1α [56]. In colon cancer, an increase in ROS levels due to elevated expression of NOX1, induced by NF-kB activation, contributes to the adhesive capacity of tumor cells [57]. Moreover, in colon cancer, increased expression of NOX2 is related to the negative regulation of the MAPK signaling pathway and an increase in proteolytic activity through elevated levels of MMP7 [58]. In gallbladder cancer have been reported an increased expression of NOX1 in cancer-associated fibroblasts, correlating with an invasive phenotype and poor prognosis [59]. In gastric cancer, an increase in NOX2 expression is linked to enhanced invasiveness of tumor cells [60]. In renal cell carcinoma subjected to hypoxic conditions, heightened NOX4 activity has been documented, contingent upon the downregulation of MAPK. This culminates in heightened production of interleukin-6 (IL-6) and interleukin-8 (IL-8), which enhance invasion of tumor cells [61]. Additionally, high expression of NOX4 in gastric cancer is associated with positive regulation of MMP7 and increased invasiveness of tumor cells [62]. The specific function of each NOX in different tumor models is detailed in Table 1. It is worth noting that the vast majority of these studies correlate NOX expression with aggressive characteristics; however, very little is known about the mechanism by which NOX-dependent ROS production modulates this malignancy. Moreover, the lack of data from different experimental conditions or patient samples makes it difficult to generalize these findings, and further studies are needed to validate these results across diverse cancer types.

**Table 1:** Invasion-related traits associated with NOXs expression and function

NOX protein	Cancer type	Associated characteristic	Reference
NOX1	Colon cancer	NF-kB promotes the expression of NOX1 and ROS levels, thereby promoting the adhesive capacity of tumor cells	[57]
	Gallbladder cancer	Increased NOX1 expression in cancer-associated fibroblasts correlates with increased cellular invasiveness and poor prognosis	[59]
	Colon Cancer	NOX1 contributes to the formation of invadopodia through the production of ROS. Its activation is dependent on the phosphorylation of NoxA1, Tks4, and Tks5 mediated by c-Src	[43, 44, 46]
NOX2	Colon Cancer	Elevated levels of ROS, dependent on increased expression of NOX2, downregulate the MAPK signaling pathway, leading to an upregulation in MMP7 production	[58]
	Gastric cancer	Increased expression of NOX2 is related to an increase in the invasiveness of tumor cells	[60]
	Cervical cancer	TGF-β1 induces the upregulation of NOX2, leading to increased expression of EMT markers	[63]
	Urothelial carcinoma	TGIF induces an increase in phosphorylation of PI3K/AKT, leading to an increase in NOX2-dependent ROS levels, thereby enhancing the invasiveness of tumor cells	[65]
NOX4	Kidney cancer	Under hypoxic conditions, a negative regulation of MAPK is generated, which is associated with increased NOX4 activity, promoting invasion through the secretion of IL-6 and IL-8	[61]
	Gastric Cancer	Overexpression of NOX4 associated with positive regulation in MMP7 production and increased invasiveness	[62]
	Cervical cancer	TGF-β1 induces the upregulation of NOX4, leading to increased expression of EMT markers	[63]
	Lung cancer	TGF-β positively regulates NOX4 expression through NF-κB activation, inducing an increase in ROS levels, thereby regulating the expression of EMT markers such as Vimentin and Snail, while decreasing the expression of E-cadherin	[64]
	Glioblastoma	TGF-β1 induces up-regulation of mRNA and protein levels of NOX4, leading to increased production of ROS. This activation subsequently triggers the PI3K/AKT/HIF-1α axis, resulting in increased expression of EMT markers such as Vimentin and N-cadherin	[66]
NOX5	Colon cancer	Associated with increased motility because it regulates integrin-linked kinase signaling pathways	[51]
	Breast cancer	NOX5 expression modulated by STAT5A, and NOX5 depletion decreases invasiveness	[52]
	Prostate cancer	Increased levels of ROS by NOX5 induces activation of HIF1α and levels of MMP14, increasing the invasiveness and proteolytic capacity of tumor cells	[53, 54]
DUOX1	Lung cancer	Induced increase of molecular marker associated to EMT through silencing of DUOX1	[49]
DUOX2	Colon cancer	Increased DUOX2-dependent ROS induces the expression of molecular markers associated with EMT	[50]

### NOXs and invasion-related signaling pathways: a feedback loop of aggressiveness

**Fig. 3:**
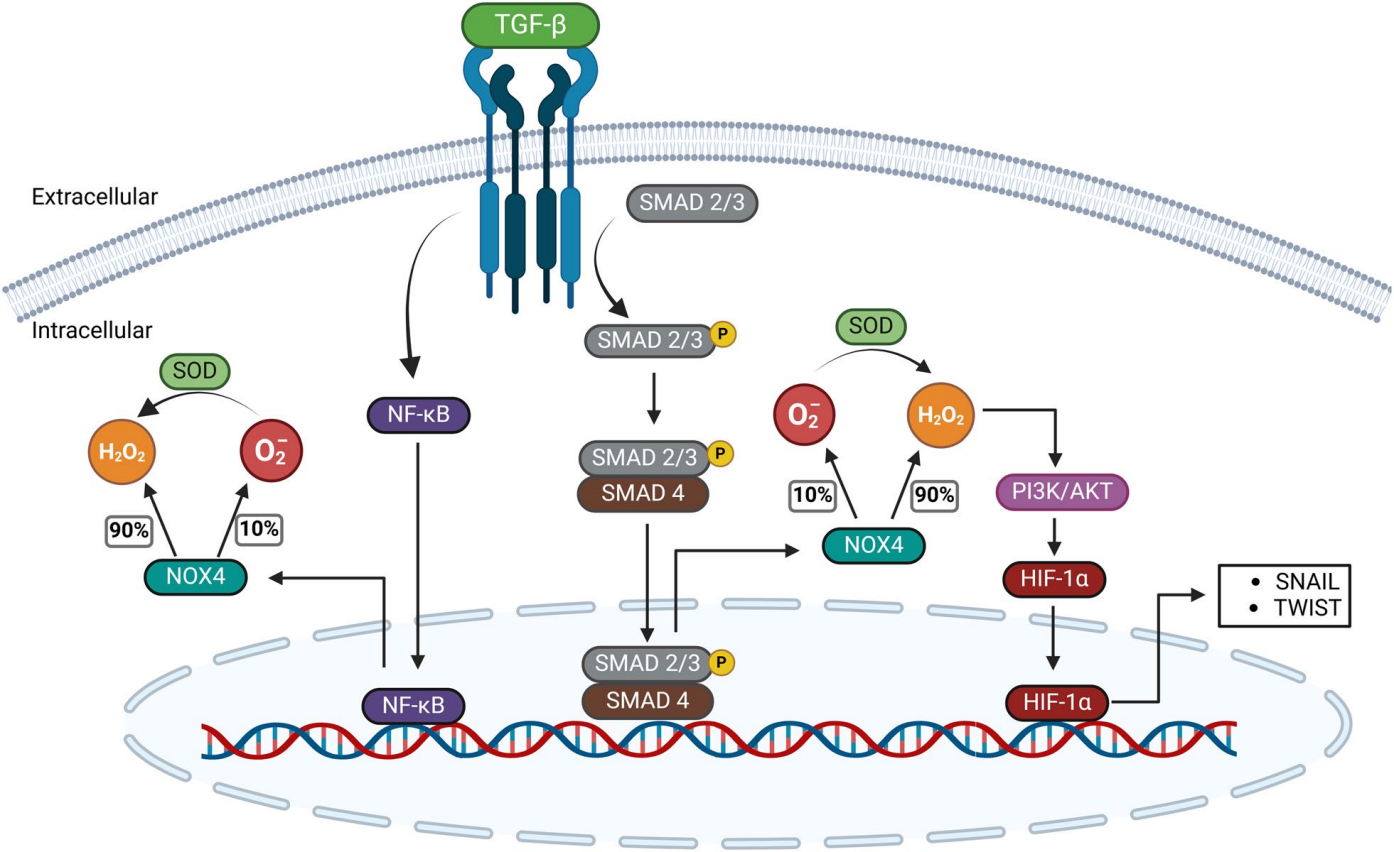
Activation and function of NOX4 in epithelial-mesenchymal transition (EMT). Mechanism by which NOX4-dependent ROS production is activated by TGF-β1 signaling and its contribution to the regulation of EMT. NOX4 expression is upregulated by TGF-β1 through the activation pathway SMAD or NF-κB, which enhances H_2_O_2_ production. NOX4 can produce O₂⁻ using 10% of the electron flow, which is rapidly converted to H₂O₂ with the help of SOD, or it can directly generate H₂O₂ using the remaining 90% of the electron flow. The figure shows how these ROS affect key EMT markers by modulating signaling pathways such as the PI3K/AKT/HIF-1α axis, increasing the expression of mesenchymal markers like Vimentin and N-cadherin, while decreasing the epithelial marker E-cadherin, thereby enhancing cancer cell invasiveness

Studies have demonstrated that tumor microenvironment molecules modulate EMT, and it has been described that they induce NOXs-dependent ROS production. It has been determined that TGF-β1, triggers an increase in ROS levels leading to the upregulation of NOX2 and NOX4. This, in turn, causes a positive upregulation of molecules related to EMT in cervical cancer cells [63]. A similar effect has been observed in lung cancer cells, where TGF-β positively regulates NOX4, inducing an increase in ROS levels mediated by the activation of NF-κB, which enhances the expression of NOX4, thereby enhancing Vimentin and Snail function and decreasing E-cadherin expression [64]. These results are consistent with those published in urothelial carcinoma cells, where overexpression of Transforming Growth Interacting Factor (TGIF) induces superoxide generation from NOX2/p67phox by blocking the SMAD signaling pathway, which is previously activated by TGF-β, thereby activating alternative the PI3K/AKT pathway, thereby increasing the invasive potential of the carcinoma cells [65] (Fig. 3). In glioblastoma cells, it has been observed that TGF-β1 induces over-regulation of mRNA expression and protein levels of NOX4 through the SMAD pathway, resulting in an increase of ROS production. This process induces the activation of the PI3K/AKT/HIF-1α axis, contributing to the EMT by increasing the expression of markers such as Vimentin and N-cadherin, while decreasing the expression of E-cadherin [66] (Fig. 3). This is consistent with other studies that detail how the stabilization of HIF-1α induces increased expression of SNAIL and TWIST, which leads to elevated production of Vimentin and N-cadherin and decreased expression of E-cadherin during EMT [67, 68]. In summary, the increase in mRNA levels and subsequent rise in NOX4 protein levels are induced by the activation of canonical and non-canonical pathways triggered by TGF-β through its interaction with the TGF receptor. Moreover, it has been described that Epidermal Growth Factor (EGF), through its receptor, induces ROS production via the activation of NOX proteins, thereby activating various signaling pathways involved in tumor cell invasion processes [69, 70]. The role of NOX proteins in invasion is covered in detail in Table 1, which outlines characteristics associated with NOX proteins and the types of cancer studied. However, the table does not include information on NOX3's involvement in tumor cell invasion. This omission is because NOX3 is known to be primarily expressed in embryonic tissues and, in adult tissues, is primarily located in the inner ear, where its presence leads to increased ROS levels, associated with hearing loss [71, 72]. Furthermore, there is evidence of low expression of NOX3 in brain and lung cells [73, 74].

In summary, NOX proteins play a crucial role in regulating various processes through the increase of reactive oxygen species (ROS). Among these processes is the remodeling of the cytoskeleton, achieved through the interaction of ROS with cofilin and G-actin, modulating the polymerization and depolymerization of actin filaments [75–78]. Additionally, NOX proteins dependent ROS interact with actomyosin, regulating its disassembly [79]. This occurs because ROS oxidize cysteine residues in proteins involved in the regulation of cytoskeleton dynamics [76, 80, 81]. Furthermore, NOX proteins are involved in the regulation of signaling pathways such as MAPK and the activation of transcription factors, including HIF-1α [61]. NOX proteins may also be subject to regulation through ROS production, thereby influencing the tyrosine phosphatase protein PEST, which, in turn, regulates the activation of Src proteins [47]. Moreover, NOX proteins participate in the activation and increased expression of MMPs in tumor cells with an invasive phenotype [39, 53]. In EMT, the activation of NOX proteins is dependent on extracellular inputs such as TGF-β1, which induces an increase in NOX-dependent ROS and the expression of EMT markers. In conclusion, NOX proteins are of great importance in the formation of the invasive phenotype in cancer cells, primarily due to their localized ROS production, which aids in the regulation of different signaling pathways. However, the various mechanisms of activation and regulation of these proteins in the invasion of cancer cells still need to be elucidated.

## Conclusion

NOX proteins play a critical role in cancer invasion through the generation of reactive oxygen species (ROS) and modulation of diverse signaling pathways. Despite significant advancements, further research is necessary to fully comprehend the activation, regulation, and interactions of NOX proteins in cancer invasion. Investigating the distinct roles of individual NOX isoforms across various cancer types and their contributions to different stages of the metastatic process are pivotal for future studies. Additionally, exploring the potential of targeting NOX proteins for therapeutic interventions in metastatic disease, alongside developing advanced imaging techniques and preclinical models, will be essential for translational research efforts. A deeper investigation into the precise mechanisms by which NOX proteins are regulated and their contributions to tumor progression and metastasis is essential. There is also a limited understanding of how the various isoforms of NOX proteins might interact with other cellular pathways involved in cancer cell migration and invasion. Ultimately, deepening our understanding of NOX-mediated mechanisms in cancer invasion holds promise for identifying novel therapeutic targets and enhancing treatment strategies against metastatic disease.

## Data Availability

Not applicable.

## Abbreviations

Arp2/3: Actin-Related Proteins 2/3
DUOX: Dual oxidase
EMT: Epithelial-Mesenchymal Transition
HIF-1α: Hypoxia-inducible factor 1α
IL-6: Interleukin 6
MMP: Matrix metalloproteinase
NOX: NADPH oxidase
NoxA1: NADPH oxidase activator 1
NoxO1: NADPH oxidase organizer 1
PI3K: Phosphoinositide 3-kinases
POLDIP2: Polymerase δ-interacting protein 2
ROS: Reactive oxygen species
TGF-β: Transforming growth factor β
TGIF: Transforming growth interacting factor
Tks: Tyrosine kinase substrate
WASP: Wiskott-Aldrich Syndrome Protein
